# Review of Coagulation Technology for Removal of Arsenic: Case of Chile

**Published:** 2006-09

**Authors:** Ana María Sancha

**Affiliations:** Department of Civil Engineering, Division of Water Resources and Environment, University of Chile, Blanco Encalada 2002, Santiago, Chile

**Keywords:** Arsenic, Arsenic removal, Coagulation technology, Drinking-water, Chile

## Abstract

Coagulation technology has been used since 1970 in northern Chile for removing arsenic from drinking-water. This experience suggests that coagulation is an effective technology for the removal of arsenic. It is currently possible to reduce arsenic from 400 μg/L to 10 μg/L at a rate of 500 L/sec, assuming pH, oxidizing and coagulation agents are strictly controlled. The Chilean experience with the removal of arsenic demonstrates that the water matrix dictates the selection of the arsenic-removal process. This paper presents a summary of the process, concepts, and operational considerations for the use of coagulation technology for removal of arsenic in Chile.

## INTRODUCTION

The presence of hazardous concentrations of arsenic in drinking-water and the serious health effects this situation is causing to untold hundreds of millions of people across the planet, have led the World Health Organization (WHO) to recommend that the maximum concentration of arsenic in human drinking-water not exceed 10 μg/L. Researchers in many countries are studying to identify the most feasible technologies for the removal of arsenic in their particular situations. Some removal systems recommended in the international water market involve advanced or emerging technologies which generally require extensive pre-treatment processes and/or very high construction, operation and maintenance costs. For many affected populations, neither they nor their governments are able to afford such expensive investments in infrastructure.

Chile, a small but emerging nation with significant arsenic exposure through water, has faced the challenge of removal of arsenic with large-scale water-treatment plants since the 1970s and has developed a strategy using the conventional technology which is both very effective and relatively inexpensive to build, operate, and maintain.

This study presents a summary of the Chilean experience in removal of arsenic from water, including an overview of the problems and variables involved and a discussion of our investigations and results at the level of large-scale water-treatment facilities.

## BACKGROUND

**Fig. 1.**
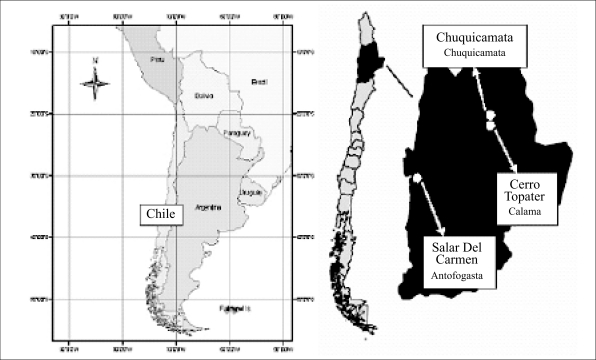
Name and location of water-treatment plants for removal of arsenic, Chile

Chile, located along a 4,320-km strip in southwestern South America (Fig. 1), with a population of approximately 15,400,000, has extensive experience in the removal of arsenic from drinking-water supplies (1, 2). Due to the particular geological characteristics of Chile and its intensive mining activity, many water sources in the northernmost area and central zones of the country are contaminated with arsenic (3).

In the late 1960s, it became evident that the consumption of water from the Toconce River—with concentrations of arsenic in the range of 600–900 μg/L—was causing serious problems for residents of the northern zone (4). The Chilean Government commissioned a study by German researchers from Berkefeld Filter concerning the removal of this contaminant from drinking-water. Working together with Chilean colleagues, these researchers ascertained the parameters required to remove arsenic from water by means of coagulation (5). The first plant was built and began operations in 1970. Four arsenic-removal plants (Table 1) that use the same process have been built in the northern zone in subsequent years (6–9).

**Table 1.** Water-treatment utilities for removal of arsenic in Chile. Chuquicamata is owned by a mining company and has limited availability of public data

Utility	Capacity (L/sec)	Water source	Arsenic range (μg/L)
Salardel Carmen Complex*			
Old plant (1970)	500	Toconce	600–900
		Lequena	150–350
New plant (1978)	520	Quinchamale	100–250
		Siloli Polapi	<50
Cerro Topater* (1978)	500	Toconce	600–900
		Lequena	150–350
		Quinchamale	140–250
Chuquicamata* (1989)	210	Colana	70–90
		Inacaliri	80–90
Taltal** (1998)	32	Agua Verde	60–80

*Surface water;

**Groundwater

Experience has confirmed that the coagulation process is a good device for both quality of water (Table 2) and volumes of water to be treated. Recently, the WHO identified the coagulation process as being the most appropriate technology to remove arsenic in large volumes (10). Numerous researchers are currently investigating the coagulation process, and their results indicate that further improvements are viable (11–16). Some new knowledge became apparent during Chile's long experience with full-scale arsenic-removal treatment plants.

**Table 2.** Quality of water in the northern zone of Chile. The process only removes arsenic. Other parameters are essentially the same in the effluent

Parameter	Unit	Surface water (range)	Groundwater (range)
PH		8.0–8.4	7.0–8.0
Total disolved solids	mg/L	700–800	730–790
Arsenic	μg/L	400–600	60–80
Sulphate	mg/L	80–100	-
Chloride	mg/L	120–140	-
Alkalinity	mg/L CaCO3	100–120	50–60
Hardness	mg/L CaCO3	130–150	350–400
Silica	mg/L SiO2	20–30	20–30
Boron	mg/L	3–4	2–5
Disolved organic carbon	mg/L	Negligible	Negligible

The Chilean drinking-water standard permitted a maximum arsenic concentration of 50 μg/L until 2004. Currently, the Chilean drinking-water standard has been modified to reach a goal of 30 μg/L in 2010 and 10 μg/L in 2015 (17). The WHO recommended that water for human consumption should not contain more than 10 μg/L (18). Currently, 99.98% of the Chilean population have access to potable water with arsenic <50 μg/L, but only 52.69% have access to potable water with arsenic <10 μg/L. To meet the new Chilean standard and the WHO guidelines, Chile will need to treat significantly more water for removal of arsenic in other zones of the country.

## REMOVAL OF ARSENIC

**Fig. 2.**
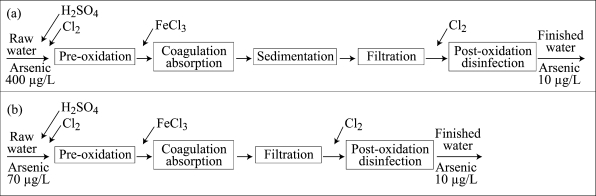
General schematic Chillean arsenic-removal treatment process: (a) Surface water and (b) Groundwater

The coagulation process consists of the addition of metal-based coagulant, such as ferric chloride (FeCl_3_), to arsenic-contaminated water. FeCl_3_ hydrolyzes in water to form positively-charged ferric hydroxide [Fe(OH)_3_]. Arsenic must be in oxidized form [As(V)] for effective removal. Thus, if any arsenite [As(III)] is present, it may be necessary to oxidize it to As(V) using chlorine as a pre-treatment process. Arsenate [As(V)] is a negatively-charged anion and sorbs to the positively-charged Fe(OH)_3_ particles or flocs. The sedimentation and filtration processes then remove arsenic particulate. A general schematic diagram of the arsenic-removal treatment process is given in Fig. 2.

The arsenic-removal system by means of coagulation in the 1970s delivered water with a residual arsenic concentration of 120 μg/L to the population of the northern Chile. The delivery concentration decreased to 50 μg/L in the 1980s and to as low as 10 μg/L in the 2000s. This increase in efficiency in the removal of arsenic has been achieved by improving the treatment-system follow-up, including control of pH and adjustment of reagent doses (19). Table 3 lists some principal arsenic-removal conditions at Salar del Carmen, Cerro Topater, and Taltal.

**Table 3.** Arsenic-removal conditions: Salar Del Carmen, Cerro Topater, and Taltal, Chile

Arsenic-removal conditions	Salar del Carmen	Cerro Topater	Taltal
Arsenic in raw water (μg/L)	400	400	70
Chemical dosage			
Oxidant (mg/L Cl_2_)	1.0	1.0	1.0
Coagulant (mg/L FeCl_3_)	56.1*	40.5*	8.0
Decantation rate (m^3^/m^2^/day)	70–75	70–75	-
Filtration rate (m^3^/m^2^/day)	143	143	150
Sludge generation (kg/day)	25–30	20–30	-
Arsenic in finished water (μg/l)	10	10	10

*H_2_SO_4_ for adjustment of pH

The coagulation processes are typically used for removing turbidity. When the same processes are used for removing arsenic from surface water, the design of the treatment system should maximize the formation of a floc with characteristics of size, cohesion, and sedimentation speed that favour stable arsenic adsorption onto it. In this way, arsenic changes from a dissolved species into a particulate species that can be separated or removed from water by means of sedimentation and filtration. In the case of groundwater, the removal process often includes only oxidation, coagulation, adsorption, and filtration. Regardless of the method of removal, the arsenic-removal process becomes a simple device for removal of suspended material. Arsenic speciation, pH, coagulant doses, and agitation speed are important parameters in this process (7, 19). Any problems that may originate in the process of floc formation and separation by sedimentation and/or filtration may limit the efficiency of removal of arsenic from water.

Recent studies demonstrated that the presence of hardness in water to be treated could favour removal of arsenic, but that some anions, especially phosphate, carbonate, and silicate, may compete with arsenic for the sorption sites, thus interfering with removal of arsenic (20, 21). Chilean water has both hardness and these competitive anions. The efficiency of the process is, thus, sensitive to the water matrix in this condition.

Quick and accurate measurement of concentrations of arsenic in water has also been a fundamental factor in improving the efficiency of the removal process. It has always been important to use the analytical method which has best responded to the requirements of the control process, to the country's economic situation, and to the abilities of its technicians. Initially, the Gutzeit method was used (22), but later, in the 1980s, the silver diethyldithiocarbamate colorimetric method was used (23), since the 1990s, hydride generation-atomic absorption spectrometry has been used (24). This has allowed more frequent adjustment of the process and the reduction of the detection limits.

Achieving higher efficiency in the removal of arsenic from water has involved greater expenditure for increasing coagulant dosage and dose automation, continuous control of pH, and more frequent monitoring of concentrations of arsenic. The FeCl_3_ dose may be reduced if pre-treatment of pH is applied (19). Greater investment, operations and maintenance costs also require additional resources to train and maintain teams of highly-qualified technicians to operate the plants.

Finally, the disposal of arsenical sludge generated during treatment of water has always been and will always be difficult due to its dangerous characteristics. In the case of Chile, the problem has been solved by carrying out this process in the desert. During the first years, this sludge was disposed of without taking any special precautions. In recent years, the process has taken place in specially-engineered sites using a geotextil as component of a landfill bottom and capping barrier systems (25)

The price of drinking-water in Chile is determined, in part, by the cost of infrastructure for treatment of water, the chemical reagents used, and the system's operation and maintenance costs. In Antofagasta, where arsenic is removed from water, the cost for a family that consumes 20 m^3^ of water per month is currently US$ 46.48, or 2.3¢ per litre. The cost for a family in Santiago, where arsenic is not removed, amounts to US$ 16.18, or about a third of the cost in Antofagasta, for the same volume of water. These figures reflect partially the relatively high cost of removal of arsenic in Northern Chile.

## FUTURE OF ARSENIC REMOVAL

Chile is making efforts to find cost-effective solutions to achieve lower levels of residual arsenic concentration in drinking-water from surface and underground water supplies. In this new scenario, another possibility that has been considered, for Northern Chile, is to replace some current sources of surface water with de-salinized sea water (26, 27). This option would only be applicable in coastal cities, but not in plants located farther from the ocean, i.e. in Salar del Carmen Plant (Antofagasta), but not in Cerro Topater (Calama).

In the central zone of Chile, where some surface waters have concentrations of arsenic in the range of 14 to 16 μg/L, modifications to the current coagulation process used for removing turbidity could meet a 10- μg/L standard. In the case of groundwater with concentrations of arsenic in the range of 20 to 80 μg/L, coagulation-filtration also would be the selected process to remove arsenic (28). Because of the afore-mentioned characteristics of water quality, adsorption processes are inefficient in the removal of arsenic. In addition, most manufacturers of sorbents do not provide regene-ration instructions.

## CONCLUSION

The Chilean experience in removal of arsenic demonstrates that the water matrix dictates the selection of the arsenic-removal process. The coagulation process has been proven to be an effective arsenic-removal process for surface and groundwater. Moreover, it has the advantage that it does not typically require excessive pre-treatment or conditioning of influents and chemicals used that are not made in Chile.

The Chilean water industry has gained significant operational experience in the removal of arsenic by coagulation and will rely on achieving a residual arsenic concentration of 10 μg/L by coagulation technology through adjustment of pH and control of coagulant dose.

The Chilean experience in the removal of arsenic at water-treatment plants demonstrates that the processes of coagulation/adsorption-sedimentation-filtration can remove arsenic up to the WHO-recommended standards for drinking-water. These processes do not require complex pre-treatment of water, but only pre-oxidation and pH adjustment. This technology for the removal of arsenic can be simplified by eliminating the sedimentation process if the conditions of water permit.

The inputs of this technology—oxidizing agent, coagulant, filtering medium—can be of local origin, and the operation of the removal system requires personnel with an intermediate level of training. The handling and disposing of the sludge generated must always be done with special precautions.

## ACKNOWLEDGEMENTS

This paper is based on studies supported by Universidad de Chile and FONDEF-CONICYT through Project FONDEF 2–24. The author is grateful for the many contributions of her students, whose cooperation made these studies possible.
